# A Transdiagnostic Approach in Psychodiagnosis: The Romanian Adaptation of the Process-Based Assessment Tool (PBAT)

**DOI:** 10.3390/bs15121697

**Published:** 2025-12-08

**Authors:** Cosmin Octavian Popa, Alina Schenk, Cristiana Manuela Cojocaru, Manuela Gyorgy, Florin Alin Sava, Ștefan Marian, Horațiu Popoviciu, Simona Szasz

**Affiliations:** 1 Department of Psychology, George Emil Palade University of Medicine, Pharmacy, Science and Technology, 540142 Targu Mures, Romania; 2 Department of Psychology, West University of Timişoara, 300003 Timişoara, Romania; 3 Department of Rheumatology, Physical and Rehabilitation Medicine, George Emil Palade University of Medicine, Pharmacy, Science and Technology, 540142 Targu Mures, Romania

**Keywords:** Process-Based Assessment Tool, psychological processes, process-based psychotherapy, Extended Evolutionary Meta-Model, anxiety, depression

## Abstract

Background: Recent research has focused on psychological constructs that maintain psychopathology, along with the constraints of single-diagnosis protocols that have contributed to the expansion of process-based psychotherapy. Therefore, the Process-Based Assessment Tool (PBAT) was developed for identifying relevant psychological processes and facilitating personalized approaches. The aim of the present study was the adaptation of the PBAT for the Romanian population. Methods: Participants (n = 637) encompassed a non-clinical and a clinical sample who filled in the PBAT. In addition, within the non-clinical group, the Patient Health Questionnaire-9 and Generalized Anxiety Disorder-7 were used for measuring the level of depression and anxiety, respectively. Results: The correlations between different PBAT processes were found in the expected direction, according to the valence of individual items. The outcomes of the network analyses revealed the centrality of several items within both samples. Also, the results of the Boruta analysis showed the predictive role of some processes in relation to anxiety and depression. Conclusions: By resembling the findings of other PBAT validation studies, the present investigation demonstrated that the instrument can be reliably applied for exploring psychological processes correlated with adaptation and maladaptation within the Romanian population.

## 1. Introduction

In contemporary clinical practice, there is a recognized need to integrate new therapeutic models relying on Cognitive Behavioral Therapy (CBT), targeting underlying dysfunctional psychological mechanisms and processes maintaining symptoms (Hofmann & Hayes, 2019).

Against this background, syndrome-based protocol psychological interventions have shown certain limitations in their long-term efficacy, particularly due to the multiple comorbidities between various types of psychopathologies that often emerge throughout the therapeutic process (Ciarrochi et al., 2024). In this context, from the perspective of the Extended Evolutionary Meta-Model (EEMM), dysfunctional psychological processes observed in psychopathology can be described in terms of psychological inflexibility, emotional dysregulation, experiential avoidance, or self-as-context (Hofmann & Hayes, 2019). The modification of these processes in psychotherapy contributes to a beneficial change from maladaptive to adaptative behaviors, correlating with the well-being of individuals (Ong et al., 2024). Here, the Process-Based Therapy (PBT) intervention can be conceptualized as dynamic, predictable, theory-driven, and multi-level in nature. The organization of these psychological processes toward personalized goals is essential for achieving therapeutic effectiveness (Hayes et al., 2020a).

According to the EEMM, the processes that differentiate between healthy psychological functioning and psychopathology can be described by the evolutionary-grounded concepts of variation, selection, retention, and context (Hayes et al., 2020b). Variation is defined as the ability to act with flexibility, in such a way that promotes adaptation to various life circumstances. In contrast, rigidity is considered a hallmark of maladaptation and distress that lies at the core of many psychological disorders (Morris & Mansell, 2018). Selection refers to the reasonable choice of a specific reaction among a broad and diverse behavioral repertoire. For example, individuals should select those behaviors that align with their personal values in order to ensure well-being in the long run (Tani, 2025). Nonetheless, unhealthy functioning is marked by unproper choices and disorganized behaviors (Blanchard et al., 2013). Retention is a key feature of psychotherapeutic outcomes, starting from the assumption that internal and external rewards are associated with the repetition of desirable behaviors and, eventually, the development of good habits (Singh et al., 2024). Context is another crucial notion for understanding the distinction between psychological robustness and dysfunction. On the one hand, heightened sensitivity to contextual aspects, along with the capacity to variably choose from a wide range of sound behaviors preserved across different situations, represent the depiction of healthy psychological adjustment. On the other hand, lack of contextual awareness portrays psychopathology defined by inflexibility, problematic action decisions, and behavioral inconsistency (Hayes & Hofmann, 2021). These processes operate across the six psychological domains, or dimensions of affect, cognition, attention, motivation, self, and overt behavior (Hayes et al., 2019). Moreover, protocol-driven approaches may reveal their limitations in the presence of psychopathological overlaps or comorbidities. In such cases, a transdiagnostic CBT intervention is recommended (Norton & Barrera, 2012; Bentley et al., 2021a, 2021b). Furthermore, an excessive focus on anxiety-specific CBT techniques in the context of anxiety disorders associated with major depressive disorder prevents the full remission of both anxiety and depressive symptoms (Gibbons & DeRubeis, 2008). Within this framework, the development of an appropriate instrument to assess progress and process-level changes in psychotherapy, particularly CBT, represented a strong requirement. Therefore, the Process-Based Assessment Tool (PBAT) represents an important tool aimed at identifying significant processes and facilitating the adaptation of psychological interventions in order to increase psychological flexibility. Thus, the items of this tool are derived from the EEMM, addressing a transtheoretical and evolutionary approach (Hayes et al., 2020b). After the PBAT initial validation (Ciarrochi et al., 2022b), the instrument was translated, adapted, and validated to be used worldwide, proving its intercultural replicability with consistent results (Talask et al., 2024; Cyniak-Cieciura et al., 2024; Larsson & Sundström, 2024). Based on previous research, the main objective of the present study is to evaluate the psychometric properties of the PBAT within the Romanian population. Accordingly, our hypotheses were the following: (1) individual PBAT items would correlate between each other in the expected directions (i.e., positive or negative), based on their valences; (2) several PBAT items would prove heightened centrality than others, emphasizing the key processes involved in adaptation and/or psychopathology; (3) similarly, several PBAT items would serve as stronger predictors for anxiety and/or depression, indicating the main processes and functional dimensions to be targeted in psychological interventions.

The Romanian adaptation of the PBAT would enhance psychological clinical practice by shifting from the traditional CBT focus on signs, symptoms, and syndromes to the underlying psychological mechanisms involved in the process of change. Moreover, the PBAT can facilitate the personalization of CBT interventions by identifying the specific maladaptive processes that lie beyond symptoms and by monitoring the process of change throughout psychotherapy. In other words, such an adaptation of the PBAT could implicitly support a paradigmatic transition in Romanian clinical practice from a standard, syndrome-based CBT framework to a modern, process-oriented one.

## 2. Materials and Methods

Participants: This study included a total of 637 participants, divided into a non-clinical group and a clinical group. The non-clinical sample consisted of 485 participants, predominantly females (430 females, 53 males, 2 other genders). The participants’ ages ranged from 18 to 62 years, with a mean age of 27.5 years (SD = 9.81). Most individuals were recruited from academic and professional environments, representing a range of educational backgrounds and occupational fields (psychology, engineering, law, education, healthcare). Inclusion criteria were as follows: aged over 18 years, ability to understand Romanian language, and voluntary agreement to participate. Exclusion criteria included incomplete or invalid questionnaire responses. The clinical sample comprised 152 individuals diagnosed with chronic medical conditions and psychiatric disorders. Of these, 111 were females and 41 were males, with an age range between 26 and 80 years (M = 56.1, SD = 13.1). The majority were married or retired, with varying levels of education. Most participants included in the clinical sample (N = 108) were diagnosed with rheumatoid diseases like osteoarthritis, rheumatoid arthritis, fibromyalgia, coxarthrosis, gonarthrosis, and chronic post-surgical pain. The remaining 44 participants were diagnosed with the following psychiatric disorders: adjustment disorder, emotional disorder, paranoid schizophrenia, neurocognitive disorder, substance use disorder, and bipolar disorder. Clinical participants were recruited from healthcare facilities with the assistance of medical staff. Inclusion criteria were the formal clinical diagnoses established by physicians specialized in rheumatology and psychiatry, respectively, along with the ability to provide informed consent. Together, the combined sample offered sufficient variability to allow comparisons between the clinical and non-clinical populations, enabling the assessment of the PBAT’s psychometric properties across diverse demographic, educational, and health-related contexts. The demographic particularities of participants are depicted in Table 1.

### 2.1. Instruments

The Process-Based Assessment Tool (PBAT) consists of 18 items, each rated on a 0–100 visual analog scale (0 = strongly disagree, 100 = strongly agree). The PBAT is a self-report measure designed to capture psychological processes of change across multiple functional domains, grounded in the EEMM of PBT (Hayes & Hofmann, 2018; Hofmann & Hayes, 2019). Unlike conventional syndrome-based instruments, the PBAT was developed to assess transdiagnostic processes that are theoretically and clinically relevant across diverse populations and therapeutic orientations. The original PBAT was created by an international panel of experts in Acceptance and Commitment Therapy, Cognitive Behavioral Therapy, Schema Therapy, Positive Psychology, and Psychodynamic Therapy (Ciarrochi et al., 2022b). Items were formulated to reflect the evolutionary principles of variation, selection, and retention, operationalized across psychological dimensions such as affect, cognition, attention, self-regulation, motivation, social connection, and health behaviors. Both positively and negatively worded items were included to differentiate adaptive functioning from maladaptive tendencies. For the present study, the PBAT was translated and culturally adapted into Romanian using forward–backward translation. The translation, adaptation, and validation on the Romanian population were possible after obtaining written permission from the developers of the original instrument (Ciarrochi et al., 2022b). The forward translation was carried over by one of the authors who is a clinical psychologist authorized to conduct domain-specific translations, which provided both field expertise in psychology and cultural adaptation skills. The backward translation was conducted by another author, also a clinical psychologist. In case of disagreements between the first two translators, a third author specialized in clinical psychology and cognitive behavioral psychotherapy provided a reconciliation solution. The iterative feedback from the authors involved in the translation process supported the conceptual and semantic appropriateness of the Romanian PBAT version. The final Romanian version of the PBAT involved a pilot testing phase, where the instrument was filled in by 10 medical students who confirmed the proper understanding of items. The instrument required minor linguistic adjustments; the subsequent empirical testing confirming the adequacy of the adapted version. The original English version, along with the Romanian version of the PBAT, are included in Appendix A. This wide response range was intentionally preserved from the original version, as it enhances sensitivity to subtle variations in clinically relevant behaviors and facilitates within-person variability analyses, which are critical for idiographic and network-based approaches (Epskamp et al., 2018). The items represent a broad spectrum of psychological processes: variation (flexibility in changing ineffective behavior), selection (using cognition to improve life, experiencing appropriate emotions, maintaining meaningful challenges), retention (sustaining strategies that are beneficial, persisting in adaptive behaviors over time), and basic needs such as autonomy, competence, social connection, and physical health (Ryan & Deci, 2017). The structure of the PBAT according to the EEMM processes and dimensions is illustrated in Table 2 and Table 3. Also, a legend linking item labels and original descriptions is provided in Appendix B.

The Patient Health Questionnaire-9 (PHQ-9) is a 9-item self-report measure widely used to assess the severity of depressive symptoms over the past two weeks (Kroenke et al., 2001). Each item corresponds to the diagnostic criteria for major depressive disorder from the DSM-IV/DSM-5, and responses are rated on a 4-point Likert scale ranging from 0 (“not at all”) to 3 (“nearly every day”). The total score ranges from 0 to 27, with higher scores indicating greater severity of depressive symptoms. The PHQ-9 has demonstrated excellent psychometric properties in both clinical and non-clinical samples, making it a reliable tool for research and practice. Indeed, a recent meta-analysis showed a pooled Cronbach alpha coefficient of 0.85 (Chae et al., 2025).

The Generalized Anxiety Disorder-7 (GAD-7) is a 7-item instrument designed to measure symptoms of Generalized Anxiety Disorder over the previous two weeks (Spitzer et al., 2006). Items are rated on a 4-point Likert scale from 0 (“not at all”) to 3 (“nearly every day”), yielding a total score between 0 and 21. Higher scores indicate greater anxiety symptom severity, with established cut-off points for mild, moderate, and severe anxiety. The GAD-7 has shown strong reliability, validity, and sensitivity to change in both epidemiological and clinical contexts (Johnson et al., 2019). The instrument has been adapted for the Romanian population, indicating good internal consistency, with Cronbach’s alpha levels of 0.92 for the clinical sample and 0.75 for the non-clinical sample (Cotiga et al., 2023).

### 2.2. Procedure

Participants were informed of the study objectives and provided informed consent before data collection. The non-clinical sample completed the PBAT individually either online or in person in university settings, while the clinical sample was recruited from patients with chronic medical conditions. Besides the PBAT, the non-clinical sample filled in the PHQ-9 and GAD-7 instruments in order to analyze the predictive potential of PBAT items reflecting adaptative and maladaptive behaviors in relation to anxiety and depression. This procedure could not have been replicated in the clinical sample since most data collected in this group were obtained from participants enrolled in past studies. Average completion time was approximately 10–15 min. All responses were anonymized to ensure confidentiality.

### 2.3. Ethical Considerations

The research protocol was approved by the Ethics Committee for Scientific Research of the George Emil Palade University of Medicine, Pharmacy, Science and Technology, under the formal decision number 3007, issued on 1 April 2024. This ethical approval was renewed by the same ethics committee in April 2025 for another 12-month interval, which covered the entire data collection phase for the present study. Prior to data collection, participants provided informed consent in writing, by signing the document, or electronic form, by submitting their demographic information and agreeing to continue filling in the online form containing the instruments used within the non-clinical sample, specifically the PBAT, PHQ-9, and GAD-7. The anonymity of responses was guaranteed, and no personally identifiable information was retained in the dataset. Data were stored securely and used exclusively for scientific purposes.

### 2.4. Statistical Analyses

Data were analyzed with R version 4.5.0 (R Core Team, 2024). All PBAT items met normality thresholds (skewness between −2 and +2, kurtosis between −7 and +7). The following statistical analyses were conducted:

1. Descriptive statistics (means, SDs, skewness, kurtosis) were computed for PBAT items and clinical outcomes (PHQ-9 for depression, GAD-7 for anxiety).
2. Pearson correlations were performed to assess relationships between PBAT items and clinical outcomes. Answers to PBAT items did not produce any missing data.
3. Network analysis was conducted for both the non-clinical and clinical samples with the bootnet package using EBICglasso estimation with the standardly employed regularization parameter of 0.50 (Epskamp et al., 2018). The regularization parameter can be adjusted to obtain either more dense network connections by lowering the parameter or to obtain a more parsimonious network by increasing the parameter. Our networks did not require any adjustment of regularization, and we maintained the standard gamma threshold of 0.50. Network centrality and stability were examined via case-dropping bootstraps with 2000 bootstrap iterations. Stability of edges was examined with non-parametric bootstrapping with 2000 iterations. We opted to use 2000 iterations as opposed to the typical 1000 iterations to increase the accuracy of the results.
4. Boruta feature selection was applied on data obtained within the non-clinical sample to identify PBAT items most strongly associated with depression (PHQ-9) and anxiety (GAD-7). The algorithm compared observed variable importance with randomly permuted shadow features, confirming significant predictors across 1000 importance runs (Kursa & Rudnicki, 2010).
5. Network invariance test (NCT) compared clinical and non-clinical subsamples in terms. The test was used to compare the structure of the two networks (network invariance) and the global strength invariance—a measure of network connectivity. A non-significant *p*-value (above 0.05) for the NCT is typically interpreted as an indication of invariance of the two compared networks (van Borkulo et al., 2023).

All statistical tests were two-tailed with significance set at *p* < 0.05. Traditional internal consistency indices (e.g., Cronbach’s α) are not conceptually appropriate, as PBAT items capture distinct functional processes rather than forming homogeneous scales. Instead, we assessed construct validity via correlations with PHQ-9 and GAD-7 in the non-clinical sample. Known-groups validity was evaluated by comparing clinical and non-clinical networks. Given the theoretical foundations of the PBAT, network modeling and feature-selection analyses (Boruta) provide more appropriate indicators of psychometric robustness than traditional trait-based metrics, as indicated within the original validation study and upcoming adaptations (Ciarrochi et al., 2022b; Cyniak-Cieciura et al., 2024; Larsson & Sundström, 2024). Together, these analyses offer coherent evidence of reliability, construct validity, and known-groups differentiation aligned with a process-based assessment approach.

## 3. Results

To estimate the networks of PBAT items in clinical and non-clinical populations, we used the bootnet package (Epskamp et al., 2018) from R (R Core Team, 2024). Specifically, we used the EBICglasso (Extended Bayesian Information Criterion Graphical Lasso) method, which is a statistical method used for estimating sparse Gaussian graphical models. It combines the graphical lasso (glasso) technique, which imposes L1 regularization to estimate a sparse inverse covariance (precision) matrix, with model selection via the Extended BIC (EBIC) criterion. We used the default tuning value of 0.50. The graphical lasso encourages sparsity by shrinking small partial correlations toward zero, effectively identifying conditional dependencies between variables. EBICglasso enhances this by systematically selecting the model with the best balance between goodness of fit and complexity, using the EBIC to penalize models with too many edges. This results in a parsimonious and interpretable network structure. To plot the networks, we used the qgraph package (Epskamp et al., 2012). The networks reported in this study are all based on Pearson correlation matrices. The correlation method is selected automatically by applying the estimateNetwork function based on the number of categories in a variable. Variables with more than seven categories are considered numerical, and Pearson correlations are applied (see documentation for function *cor_auto* in package *qgraph*; Epskamp et al., 2012).

We also applied Boruta analysis to identify which PBAT items are most important in relationships with PHQ-9 depression and GAD-7 anxiety scores within the non-clinical sample. We applied this analysis through the Boruta package in R (Kursa & Rudnicki, 2010). Boruta analysis is a feature selection method used in machine learning to identify the most important variables for predicting a given outcome. It is built around a random forest algorithm and works by comparing the importance of actual features with that of randomly permuted versions of those features, called “shadow features.” By performing this, Boruta can determine whether a feature has significantly higher importance than what would be expected by chance. Features that consistently outperform their shadow counterparts are considered truly important, while those that do not are rejected. This helps improve model performance by eliminating irrelevant or redundant variables, making the model more interpretable and efficient.

The databases included in the present study are available upon request from the corresponding author. All study variables represented a normal distribution, following the rule of skewness being inside the −2 … +2 interval, and kurtosis inside the −7 … +7 interval. Descriptive statistics of the study variables are presented in Table 4.

Additionally, Table 5 presents the Pearson correlations obtained between the PBAT items, PHQ-9, and GAD-7. Overall, PBAT items seem to be weakly to moderately related to each other. One exception is PBAT_5 (“NoMeaningfulChallenge”), which displayed a significant correlation only with PBAT_11 (“ImportantChallenge”). Some unexpected correlations appeared with anxiety and depression, such as a negative correlation with PBAT_5 (“NoMeaningfulChallenge”). Additionally, PBAT_4 (“Struggled to keep doing imp”) displayed an insignificant relationship with depression and anxiety; PBAT_10 (“Stuck to working strategies”) was positively related to depression and anxiety; PBAT_11 (“ImportantChallenge”) was not related to depression; and PBAT_15 (“ConnectToPeople”) had a positive relationship with anxiety and depression.

### 3.1. Network Stability and Centrality

**Figure 1:**
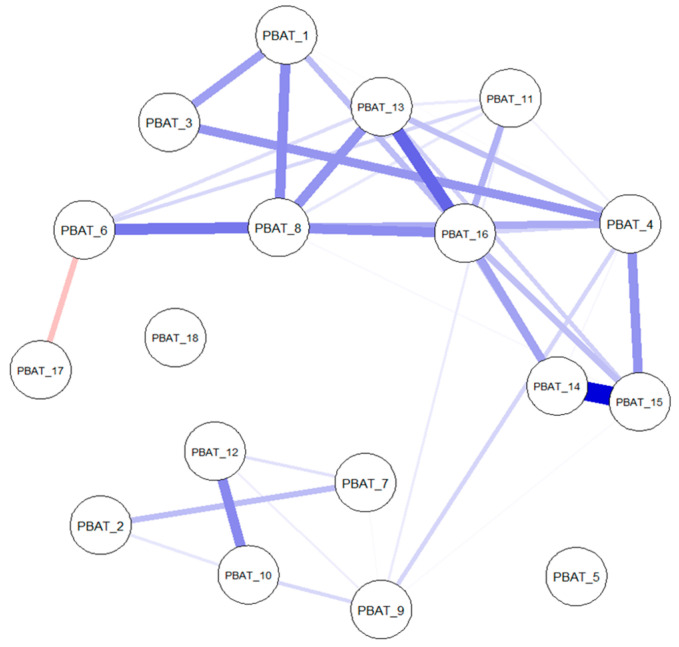
The network for the non-clinical sample.

**Figure 2:**
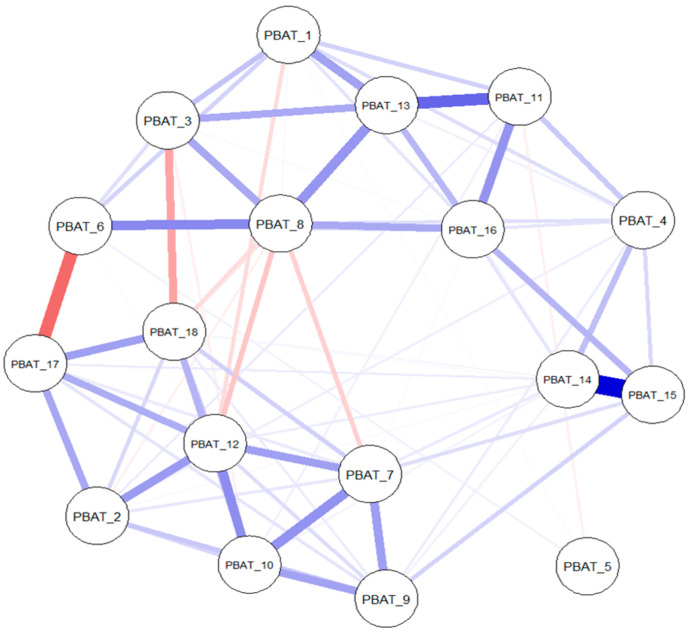
The network in the clinical sample.

The network obtained for the non-clinical sample is depicted in Figure 1. Strength centrality of this network indicated good stability levels, CS = 0.67, indicating that the correlation between original and bootstrapped strength values will drop below 0.70 only when removing 67% of the data. The network in the clinical sample is displayed in Figure 2. The suboptimal strength stability coefficient of CS = 0.20 obtained for the clinical network implies the depiction of preliminary rather than definitive results. Therefore, these centrality findings are interpreted as exploratory and should be approached with caution.

**Figure 3:**
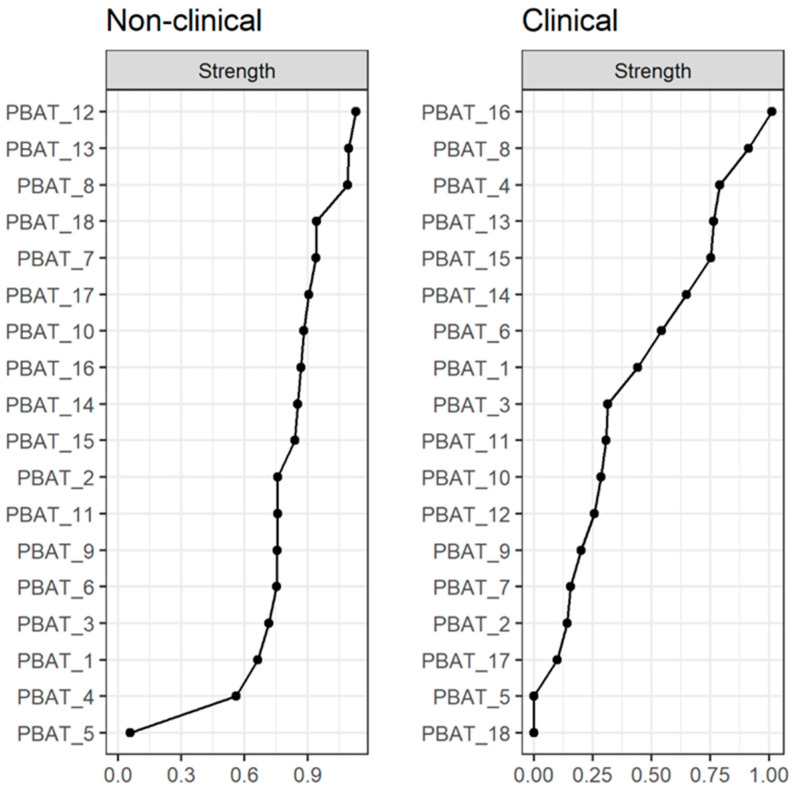
Centrality coefficients for both networks.

Centrality coefficients for both networks are presented in Figure 3. PBAT_8 (“PaidAtToImportant”) was central in both networks. PBAT_12 (“StuckUnableChange”) and PBAT_13 (“ThinkingHelpedLife”) were central in the non-clinical network, while PBAT_16 (“PersonalImport”) and PBAT_4 (“StruggledToKeepDoing”) were central in the clinical network. This indicates the possibility that some processes are more influential in individuals that have a clinical diagnosis, while others are more influential in non-clinical individuals. Stability of edges is presented in the online Supplementary Material. In the non-clinical network, most of the edges did not contain zero in the confidence intervals, while in the clinical network the majority of edges seem unstable. We will refrain from interpreting the meaning of unstable edges further in the manuscript.

### 3.2. Network Invariance

The comparison using the NCT (van Borkulo et al., 2023) returned statistically insignificant results indicating that the structure of the two networks (clinical vs. non-clinical) are invariant: M = 0.25, *p* = 0.18, and *p* > 0.05. These results point to the fact that both networks show the same structure and patterns of associations (are invariant). Additionally, there were no significant differences in the global strength of the two networks, S = 3.46, *p* = 0.20, and *p* > 0.05, indicating that nodes in the two networks have comparable levels of centrality at the chosen alpha level. Mean of the strength indices in the non-clinical sample was m = 7.28 and in the clinical sample was m = 3.81. Based on van Borkulo et al. (2023) recommendations, in a network with 20 nodes, power was acceptable under all circumstances simulated in their study. As per this recommendation, our sample size (N = 637) seems to be adequate to apply the NCT to networks of 18 nodes. This indicates that the insignificant result is not an issue of statistical power.

*Boruta analysis*. A Boruta algorithm was applied to determine what PBAT processes are more important in a relationship with anxiety and depression, based on the data obtained from the non-clinical group. PBAT_18 (“NoOutletForFeelings”) seems to be the most important process for both conditions. It is possible that participants that endorse this process will also have high levels of anxiety and depression. Additionally, processes PBAT_7 (“ThinkingGotInWay”) and PBAT_17 (“HurtHealth”) were also important for both anxiety and depression. PBAT_12 (“StuckUnableChange”) seems to be important only in relationship with anxiety, while PBAT_3 (“ExperienceRangeEmotions”) had higher levels of importance related to depression. Some processes were identified to be irrelevant for anxiety, such as PBAT_11 (“ImportantChallenge”) and PBAT_4 (“StruggledToKeepDoing”), and others were found irrelevant for depression, such as PBAT_15 (“ConnectToPeople”), PBAT_11 (“ImportantChallenge”), PBAT_1 (“AbleToChangeBehavior”), and PBAT_4 (“StruggledToKeepDoing”). Additionally, PBAT_5 (“NoMeaningfulChallenge”) was a predictor of anxiety, while only being tentative for depression. At the same time, despite that PBAT_15 (“ConnectToPeople”) was rejected as a predictor for depression, it was found to be tentative for anxiety. The results of the Boruta analysis, such as item importance and decision, are presented in Table 6. We complemented the Boruta analysis with multiple regression analyses to estimate how much of the variance of GAD-7 and PHQ-9 is explained by individual PBAT items. Although the Boruta algorithm has confirmed the importance of many items, the individual contribution to the explained variance seems to be low, between 0.00 and 0.04.

## 4. Discussion

As a tool for assessing behavioral features involved in adaptation and maladaptation, the PBAT represents an instrument based on individual items corresponding to the processes pointed out in the EEMM. The aim of the present study was to emphasize the psychometric qualities of this instrument within the Romanian population. Consequently, network analyses were carried over for each group, reflecting similar patterns of associations in terms of the PBAT’s internal structure. Nonetheless, due to the exploratory nature of the data obtained based on the network included in the clinical sample, these findings should be interpreted with caution. Additionally, a Boruta analysis was applied to indicate the relevance of PBAT items in relation to anxiety and depression symptoms. Adaptive processes showed expected negative associations with distress, while maladaptive processes converged positively, supporting convergent/discriminant validity within a process-based framework.

### 4.1. Results of Correlational Analyses

First, despite representing separated processes, this study showed associations between different PBAT items, consolidating the role of the EEMM framework for understanding human behavior in different circumstances. Overall, these results resembled the pattern found in similar investigations, showing positive correlations between items with the same valence (Ciarrochi et al., 2022b; Sanford et al., 2022).

For example, item “AbleToChangeBehavior” (PBAT_1) was found to be directly proportional with several positive selections item such as “ExperienceRangeEmotions” (PBAT_3), “PaidAtToImport” (PBAT_8), “ImportantChallenge” (PBAT_11), and “ThinkingHelpedLife” (PBAT_13). This points to an existing correspondence between healthy variation and the ability to identify workable affective, attentional, cognitive or motivational strategies, along with deliberately engaging in those behaviors (Ciarrochi et al., 2022a). Similarly, the negative selection item defining a cognitive dimension “ThinkingGotInWay” (PBAT_7) was significantly related to other negative selection items referring to the social connection (“HurtConnect”; PBAT_2), motivational (“Complying”; PBAT_9), and affective (“NoOutletForFeelings”; PBAT_18) dimensions, along with the negative variation item (“StuckUnableToChange”; PBAT_12). In other words, this is consistent with the idea that psychological rigidity/ inflexibility involves multiple domains, creating a picture of interrelated processes potentially leading to maladaptation (Levin et al., 2014). Moreover, several negative items established positive correlations with depression and anxiety levels, including “HurtConnect” (PBAT_1), “ThinkingGotInWay” (PBAT_7), “Complying” (PBAT_9), “StuckUnableToChange” (PBAT_12), “HurtHealth” (PBAT_17), and “NoOutletForFeelings” (PBAT_18). This pattern pointed to the links between different processes involving the cognitive, affective, social, and even physical health dimensions and the experience of emotional distress, as confirmed by previous investigations (Habibović et al., 2025; Orouji et al., 2022). Likewise, mirroring these outcomes, some positive items were inversely associated with depression and anxiety, indicating the adaptive role of processes such as emotional acceptance (“ExperienceRangeEmotions”; PBAT_3), mindfulness/present moment awareness (“PaidAtToImportant”; PBAT_8), and functional thinking style (“ThinkingHelpedLife”; PBAT_13) (Landi et al., 2022). However, some items showed unexpected correlations with distress levels. For example, “NoMeaningfulChallenge” (PBAT_5) and “ConectToPeople” (PBAT_15) were correlated with both depression and anxiety in opposite directions. We consider that this suggests the dynamic characteristics of these processes as a function of one’s goals in certain contexts (Ciarrochi et al., 2024). At the same time, it could also reveal the willingness to pursue personal values and engage in social connections in the general population, especially when confronted with psychological challenges. Presumably, these unpredicted outcomes are compatible with the functional contextualism framework proposed in PBT, pointing to an increased use of these behavioral strategies as coping mechanisms within the studied sample. Moreover, this shows the contextual sensitivity of the PBAT by highlighting cultural particularities that might influence the overall representation of target processes correlated with anxiety and depression. Specifically, considering the portrayal of cultural dimensional, the Romanian population is known for presenting a tendency toward avoidance of uncertain contexts. In this regard, unclear personal values and unworkable action, reflected through “NoMeaningfulChallenge” (PBAT_5), can serve as a relevant stressor associated with emotional difficulties. Correspondingly, as illustrated by its collectivistic inclinations, Romanian citizens are more likely to value the sense of community and to return to social interactions as an emotional management strategy. We assume this depiction relates to the link between “ConectToPeople” (PBAT_15), anxiety, and depression (Hofstede et al., 2010).

### 4.2. Outcomes of the Network Analyses

The network analysis performed in this study revealed invariant associations between the nodes across the non-clinical and the clinical samples. Specifically, the centrality of the positive selection item “PaidAtToImportant” (PBAT_8) emerged in both networks, highlighting the critical role of attentional processes in terms of psychological functioning, often conceptualized as mindfulness or present-moment awareness (Aizik-Reebs et al., 2021; Hayes & Hofmann, 2021). Likewise, the positive selection item “ThinkingHelpedLife” (PBAT_13) was central in the non-clinical network, pointing to the function of cognitive processes facilitating an adaptive thinking style. This is consistent with processes-based models that outline the pivotal function of modifying negative and dysfunctional thought patterns for promoting well-being (Wall & Lee, 2025; Moorey, 2023). While several items indicated an increased significance within the clinical sample network, including “StuckUnableChange” (PBAT_12), “StruggledToKeepDoing” (PBAT_4), and “PersonalImport” (PBAT_16), the explanations of these results are merely tentative, given that these are preliminary findings requiring replication. Nonetheless, these probing outcomes could reflect an increased concordance with the psychological flexibility model, placing rigidity at the core of psychological malfunctioning (Faustino et al., 2023).

We believe that these results mirror the potential components to be integrated within psychological interventions. Given the diversity of psychopathologies reflected in our clinical sample, including emotional and psychotic disorders, we consider that the improvement of motivational processes, particularly emphasized through the use of values-based approaches, could be an important psychotherapeutic goal across multiple diagnoses (Paliliunas, 2022; Rahal & Gon, 2020; Wilson et al., 2004).

### 4.3. Results of the Boruta Analysis

The Boruta analysis revealed the main predictors of clinical outcomes, particularly anxiety and depression, in the non-clinical sample. Similarly to other studies, negative selection behaviors were mostly associated with anxiety and depression (Cyniak-Cieciura et al., 2024; Larsson & Sundström, 2024). Compared with the predictive values of PBAT items for sadness and anxiety obtained by Ciarrochi et al. (Ciarrochi et al., 2022b), we identified different predictive values for those items in our sample. Additionally, the majority of items, excluding PBAT_4 (“StruggledToKeepDoing”) and PBAT_10 (“StuckToStrategies”), were found relevant for the investigated clinical symptoms, which resembled the outcomes of the original validation study (Ciarrochi et al., 2022b). Specifically, the primary predictor for both anxiety and depression was PBAT_18 (“NoOutletForFeelings”), meaning that individuals experiencing distress will resort to the avoidance of negative feelings. Our result reinforces the need to expand emotional flexibility by encouraging individuals to develop acceptance and downregulate their emotions throughout the psychotherapeutic process (Ong et al., 2024). Alongside negative selection behaviors, “StuckUnableChange” (PBAT_12) was identified as the second important process in relation with anxiety. This process represents the core of evolutionary change and adaptation, facilitating the selection and retention of healthy behaviors (Hayes et al., 2020b). Therefore, by expanding the repertoire of new responses at the cognitive, emotional, and behavioral levels, it could be considered to overcome threatening situations. In relation with depression, “StuckUnableChange” (PBAT_12) was a little more distal but still among the most relevant ten items. Furthermore, “ThinkingGotInWay” (PBAT_7) and “HurtHealth” (PBAT_17) were significant predictors for clinical outcomes. This highlights the importance of cognitive processes for clinical outcomes. Several investigations found a strong association between cognitive processes, like cognitive fusion, psychological inflexibility, and emotional distress (Barrera-Caballero et al., 2021; Tavakoli et al., 2019). Therefore, dealing with unhelpful ways of thinking by using diffusion techniques would improve the emotional state of individuals, allowing them to become more aware of their psychological experience and to act according to their life values.

Among the positive selection behavior found in the top 10, “ExperienceRangeEmotions” (PBAT_3), “HelpHealth” (PBAT_6), “StuckToStrategies” (PBAT_10), and “ThinkingHelpedLife” (PBAT_13) are included. On the one hand, these outcomes could reflect the need for further refinement of items adaptation for the Romanian population considering cultural particularities. On the other hand, despite the fact that these results refer to a group without psychopathology, these items could indicate salient processes to be included in psychotherapeutic approaches, considering idiographic characteristics for tailoring such protocols when treating emotional disorders. Nonetheless, some PBAT items like “HurtConnect” (PBAT_2), “NoMeaningfulChallenge” (PBAT_5), and “StruggleConnectMoments” (PBAT_14) were less significant in relation to emotional disorders in our non-clinical sample. Once more, these items emphasize the cultural context of the Romanian population, along with the increased sensitivity to some of these processes. Although it has been identified in other studies as an important predictor for clinical outcomes, “StruggledToKeepDoing” (PBAT_4) was rejected as a predictor in our analysis. The outcomes of our investigation could be compared with the results of the Boruta analysis obtained in other studies. For example, the main predictors of anxiety and depression in our sample were also identified as determinants of emotional disorders in the Polish population (Cyniak-Cieciura et al., 2024). Thus, “NoOutletForFeelings” (PBAT_18) was found to serve as the most important predictor for both anxiety and depression. Furthermore, “StuckUnableChange” (PBAT_12), “ThinkingGotInWay” (PBAT_7), “ThinkingHelpedLife” (PBAT_13), “Complying” (PBAT_9), and “HelpHealth” (PBAT_6) were the main processes involved in the occurrence of anxiety. For affective disorders in general, the strongest similar processes were the following: “StuckUnableChange” (PBAT_12), “ThinkingGotInWay” (PBAT_7), and “Complying” (PBAT_9). Analyzing the top ten processes related with anxiety and depression in the Spanish population, we observed that over a half of the processes were present as predictors for the same clinical symptoms in our sample (Goicoechea et al., 2024). The items “NoOutletForFeelings” (PBAT_18), “StuckUnableChange” (PBAT_12), “ThinkingGotInWay” (PBAT_7), “HurtHealth” (PBAT_17), and “ThinkingHelpedLife” (PBAT_13) overlap for both anxiety and depression, emphasizing the transdiagnostic value of the PBAT. The observed similarities could be explained by the increased uncertainty avoidance that characterizes these three countries, leading individuals to experience rigid beliefs and behaviors (Hofstede, 2001). Furthermore, a large number of negative behavior selections found as predictors of psychopathology in the Swedish population were similarly identified in the Romanian sample (Larsson & Sundström, 2024). For instance, at the selection level, the same four negative behaviors, namely “ThinkingGotInWay” (PBAT_7), “NoOutletForFeelings” (PBAT_18), “Complying” (PBAT_9), and “HurtHealth” (PBAT_17), predicted emotional disorders in both groups. Depicting the variation process, “StuckUnableChange” (PBAT_12) correlated with depressive and anxious symptoms in both samples. Once again, these findings emphasize the transcultural nature of the PBAT for identifying adaptive and maladaptive behavioral processes.

Despite several discrepancies between the individual values of the processes, overall, the Boruta analysis underlined the feasibility and usability of the instrument to measure the occurrence of behavioral strategies in the general population. Generally, our findings support the use of the PBAT as a transdiagnostic tool, pointing to the processes involved in multiple psychopathologies, making it a feasible instrument across different psychodiagnoses. Moreover, the identification of key adaptation and maladaptation processes could maximize the efficiency of psychotherapy by providing the possibility of working in a highly personalized, tailored way, while also enhancing the employment of resources along this path. However, it is worth noting that the results of the Boruta analysis were mostly exploratory, which could explain the unexpected pattern of item salience in relation to anxiety and depression. In this way, there is an increased need for future investigations replicating these analyses and further exploring the relation between PBAT items and psychopathology.

### 4.4. Limitations and Future Research

There are several limitations that could hinder the interpretability of the outcomes in the present study. First, the fact that it was not possible to apply the PHQ-9 and GAD-7 within the clinical sample prevented us from analyzing the concurrent validity of the PBAT based on this data and extrapolating the obtained results to the clinical population. Second, the transversal design used in our research does not allow the evaluation of the test–retest reliability of the Romanian PBAT version. Third, the translation process of the PBAT implemented in our investigation did not involve an expert review phase, which could reflect a constraint related to the linguistic adaptation of the instrument. Therefore, the current adaptation of the Romanian PBAT version could involve a specialized semantic review conducted by an expert committee for consolidating our findings related to proper comprehension and cultural suitability. Another constraint, which could limit the generalizability of our results to a broader Romanian population, consists in the heterogenous and small sample of the clinical sample along with the fact that the non-clinical sample included participants mainly from academic and professional contexts. A further limitation concerns the low centrality–stability coefficient in the clinical network, which constrains the interpretability of clinical centrality estimates and justifies our decision to treat them as exploratory and with caution. As a result, future studies should replicate our findings, involving homogenous measures and statistical analyses across samples with different characteristics, a test–retest reliability assessment, as well as larger sample sizes for confirming the accuracy of the present Romanian PBAT adaptation.

## 5. Conclusions

In conclusion, the present study confirms the successful Romanian adaptation of the PBAT, demonstrating its potential to assess key adaptive and maladaptive behavioral processes. Our findings are in accordance with the EEMM components of variation, selection, retention, and context, highlighting the central role of different dimensions of psychological functioning. As a culturally sensitive tool, the PBAT proves to be a valuable instrument for the idiosyncratic evaluation of adaptation processes, along with the development of targeted psychotherapeutic interventions.

In clinical practice, the PBAT may represent a valid tool for assessing specific behavioral processes, aiding its usefulness for monitoring therapeutic progress. Therefore, the Romanian version of the PBAT can successfully be integrated into specialized training programs, especially for practitioners embracing an approach based on CBT or ACT.

In line with our findings, future investigations could involve a uniform assessment process across different study groups, refining the translation and adaptation procedures and including rigorous statistical analyses for validating the psychometric properties of the Romanian version of the PBAT.

## Figures and Tables

**Table 1:** Sociodemographic characteristics.

	Non-Clinical Sample (N = 485)		Clinical Sample (N = 152)		Overall (N = 637)	
Age (mean, SD)	27.5	9.81	56.1	13.1	34.3	16.2
Gender (N, %)						
Female	430	88.7	111	73	542	85.2
Male	53	10.9	41	26.9	94	14.7
Other	2	0.4		2	0.3	
Education						
Professional/High school	102	21	75	49.3	177	27.8
Higher education	383	79	39	25.6	422	66.2
Middle school	0	0	38	25	38	5.9
Main diagnosis						
Adjustment disorders	-	-	11	7.2	-	1.7
Bipolar disorder	-	-	1	0.65	-	0.15
Emotional disorders	-	-	19	12.5	-	3
Paranoid schizophrenia	-	-	8	5.3	-	1.25
Chronic pain	-	-	108	71	-	17
Substance use disorders	-	-	2	1.3	-	0.3
Neurocognitive disorders			2	1.3	-	0.3

Note. N = sample size, SD = standard deviation.

**Table 2:** The correspondence between EEMM processes and individual PBAT items.

Selection Process—Positive	Selection Process—Negative	Variation—Positive and Negative	Retention—Positive and Negative
PersonalImport (PBAT_16)	Complying (PBAT_9)	AbleToChangeBehavior (PBAT_1)	StuckToStrategies (PBAT_10)
HelpHealth (PBAT_6)	HurtHealth (PBAT_17)	StuckUnableChange (PBAT_12)	StruggledToKeepDoing (PBAT_4)
PaidAtToImportant (PBAT_8)	StruggleConnectMoments (PBAT_14)		
ConnectToPeople (PBAT_15)	HurtConnect (PBAT_2)		
ExperienceRangeEmotions (PBAT_3)	NoOutletForFeelings (PBAT_18)		
ImportantChallenge (PBAT_11)	NoMeaningfulChallenge (PBAT_5)		
ThinkingHelpedLife (PBAT_13)	ThinkingGotInWay (PBAT_7)		

Note. The present table was adapted after (Ciarrochi et al., 2022b).

**Table 3:** PBAT items reflecting negative/positive behaviors mapped according to EEMM processes and dimensions.

EEMM Process	Negative Behavior	Positive Behavior
**Selection**		
Affect	NoOutletForFeelings (PBAT_18)	ExperienceRangeEmotions (PBAT_3)
Cognition	ThinkingGotInWay (PBAT_7)	ThinkingHelpedLife (PBAT_13)
Attention	StruggleConnectMoments (PBAT_14)	PaidAtToImportant (PBAT_8)
Social connection	HurtConnect (PBAT_2)	ConnectToPeople (PBAT_15)
Motivation	Complying (PBAT_9)	PersonalImport (PBAT_16)
Overt behavior	NoMeaningfulChallenge (PBAT_5)	ImportantChallenge (PBAT_11)
Physical health behaviors	HurtHealth (PBAT_17)	HelpHealth (PBAT_6)
**Variation**	StuckUnableChange (PBAT_12)	AbleToChangeBehavior (PBAT_1)
**Retention**	StruggledToKeepDoing (PBAT_4)	StuckToStrategies (PBAT_10)

Note. The present table was adapted from (Cyniak-Cieciura et al., 2024).

**Table 4:** Descriptive statistics for PBAT items’ clinical outcomes.

	Non-Clinical				Clinical			
Variable	M	SD	Skew	Kurt	M	SD	Skew	Kurt
PBAT1	75.46	21.92	−1.14	1.10	66.32	26.71	−0.77	−0.14
PBAT2	53.65	32.10	−0.13	−1.26	23.49	28.27	1.11	0.16
PBAT3	76.87	22.37	−1.14	0.80	62.04	29.01	−0.56	−0.51
PBAT4	76.74	24.66	−1.19	0.68	73.22	26.88	−0.99	0.21
PBAT5	21.57	26.50	1.39	1.07	38.29	32.34	0.31	−1.1
PBAT6	59.20	27.26	−0.31	−0.89	63.29	29.9	−0.37	−0.85
PBAT7	63.48	29.18	−0.53	−0.77	47.76	31.31	−0.1	−1.14
PBAT8	70.97	23.12	−0.76	0.02	76.91	25.14	−1.1	0.68
PBAT9	67.18	29.49	−0.77	−0.48	52.76	34.01	−0.18	−1.31
PBAT10	52.99	29.09	−0.09	−1.03	47.04	31.74	−0.08	−1.34
PBAT11	74.33	23.82	−1.09	0.76	60.46	31.88	−0.44	−0.95
PBAT12	56.56	31.22	−0.17	−1.23	39.47	30.31	0.38	−1.07
PBAT13	80.21	19.97	−1.41	2.12	77.04	24.65	−1.25	1.03
PBAT14	64.35	27.63	−0.62	−0.52	72.96	25.76	−0.99	0.33
PBAT15	71.42	27.57	−1.00	0.08	78.95	24.77	−1.43	1.79
PBAT16	81.05	18.96	−1.26	1.55	76.45	24.48	−1.05	0.37
PBAT17	51.77	33.11	−0.08	−1.34	40.07	30.89	0.38	−0.93
PBAT18	52.62	32.43	−0.04	−1.30	46.45	30.95	0.15	−1.06
PHQ-9	1.32	0.85	0.55	−0.71				
GAD-7	1.06	0.74	0.39	−0.94				

Note: M = mean, SD = standard deviation.

**Table 5:** Pearson correlations between PBAT items, PHQ-9, and GAD-7.

Variable	1	2	3	4	5	6	7	8	9	10	11	12	13	14	15	16	17	18
1. PBAT_1		−0.00	0.40 **	0.34 **	0.1	0.30 **	0.06	0.48 **	0.13	0.06	0.26 **	0.01	0.35 **	0.20 *	0.19 *	0.43 **	−0.01	−0.13
2. PBAT_2	−0.04		0.14	−0.11	0.14	−0.01	0.33 **	−0.09	0.17 *	0.27 **	−0.11	0.13	−0.22 **	−0.07	0.03	−0.1	0.12	0.11
3. PBAT_3	0.32 **	−0.16 **		0.42 **	0.13	0.25 **	−0.02	0.27 **	0.16	0.06	0.19 *	−0.03	0.26 **	0.10	0.20 *	0.28 **	−0.05	−0.20 *
4. PBAT_4	0.19 **	0.11 *	0.11 *		−0.01	0.38 **	0.02	0.49 **	0.31 **	0.22 **	0.32 **	−0.02	0.48 **	0.41 **	0.50 **	0.48 **	0.03	−0.03
5. PBAT_5	−0.07	−0.00	−0.01	−0.03		0.23 **	0.21 **	0.09	0.07	0.03	0.05	0.02	0.10	0.06	0.04	0.10	−0.09	0.03
6. PBAT_6	0.27 **	−0.20 **	0.30 **	0.04	0.08		0.06	0.53 **	0.15	0.04	0.35 **	−0.01	0.41 **	0.26 **	0.30 **	0.43 **	−0.33 **	−0.03
7. PBAT_7	−0.11 *	0.37 **	−0.22 **	0.05	−0.03	−0.20 **		−0.01	0.24 **	0.22 **	−0.00	0.28 **	−0.04	0.08	0.17 *	0.03	0.10	0.20 *
8. PBAT_8	0.30 **	−0.25 **	0.42 **	0.19 **	0.02	0.44 **	−0.32 **		0.18 *	0.08	0.36 **	−0.07	0.56 **	0.36 **	0.40 **	0.59 **	−0.12	−0.16
9. PBAT_9	−0.02	0.36 **	−0.13 **	0.13 **	−0.02	−0.13 **	0.45 **	−0.18 **		0.30 **	0.27 **	0.27 **	0.11	0.12	0.26 **	0.20 *	0.12	0.16 *
10. PBAT_10	−0.11 *	0.39 **	−0.24 **	0.03	−0.05	−0.18 **	0.51 **	−0.20 **	0.44 **		0.17 *	0.41 **	0.06	0.14	0.23 **	0.11	0.07	0.07
11. PBAT_11	0.30 **	0.07	0.19 **	0.25 **	−0.09 *	0.18 **	−0.02	0.26 **	0.03	0.05		0.09	0.35 **	0.16 *	0.16	0.42 **	−0.05	0.09
12. PBAT_12	−0.23 **	0.48 **	−0.27 **	−0.03	0.02	−0.30 **	0.51 **	−0.37 **	0.40 **	0.52 **	−0.08		−0.03	0.11	0.12	0.03	0.10	0.21 *
13. PBAT_13	0.41 **	−0.07	0.41 **	0.22 **	−0.06	0.28 **	−0.15 **	0.47 **	−0.09 *	−0.14 **	0.48 **	−0.22 **		0.49 **	0.49 **	0.63 **	−0.12	−0.06
14. PBAT_14	0.04	0.18 **	−0.04	0.27 **	0.02	−0.04	0.23 **	−0.04	0.23 **	0.20 **	0.11 *	0.21 **	0.15 **		0.70 **	0.53 **	−0.04	0.21 **
15. PBAT_15	0.05	0.16 **	−0.02	0.26 **	0.00	0.05	0.23 **	−0.01	0.25 **	0.18 **	0.15 **	0.17 **	0.12 **	0.59 **		0.52 **	0.06	0.13
16. PBAT_16	0.28 **	0.00	0.26 **	0.24 **	−0.03	0.28 **	−0.13 **	0.40 **	−0.02	−0.08	0.41 **	−0.11 *	0.42 **	0.20 **	0.26 **		−0.02	0.07
17. PBAT_17	−0.14 **	0.42 **	−0.20 **	0.05	0.02	−0.44 **	0.36 **	−0.31 **	0.32 **	0.34 **	−0.02	0.47 **	−0.14 **	0.18 **	0.13 **	−0.10 *		0.20 *
18. PBAT_18	−0.19 **	0.37 **	−0.38 **	0.04	0.06	−0.28 **	0.41 **	−0.35 **	0.33 **	0.43 **	−0.06	0.46 **	−0.28 **	0.17 **	0.15 **	−0.17 **	0.44 **	
PHQ−9	−0.21 **	0.36 **	−0.36 **	0.02	−0.12 **	−0.39 **	0.44 **	−0.35 **	0.35 **	0.40 **	−0.08	0.51 **	−0.28 **	0.23 **	0.17 **	−0.15 **	0.42 **	0.49 **
GAD−7	−0.22 **	0.32 **	−0.41 **	−0.01	−0.10 *	−0.40 **	0.44 **	−0.43 **	0.28 **	0.39 **	−0.09 *	0.47 **	−0.33 **	0.21 **	0.13 **	−0.21 **	0.45 **	0.56 **

Note: * *p* < 0.05, ** *p* < 0.01; above the diagonal space are correlations in the clinical sample and below the diagonal space are correlations in the non-clinical sample. GAD-7 = −Generalized Anxiety Disorder-7, PHQ-9—Patient Health Questionnaire-9, PBAT—Process-Based Assessment Tool.

**Table 6:** Results of Boruta analysis for the non-clinical sample.

GAD-7						PHQ-9					
Predictor	meanImp	Decision	Beta	Beta 95% CI [LL, UL]	*sr* ^2^	Predictor	meanImp	Decision	Beta	Beta 95% CI [LL, UL]	*sr* ^2^
			*R*^2^ = 0.454 **						*R*^2^ = 0.497 **		
PBAT_18	17.01	Confirmed	0.18	[0.10, 0.27]	0.02	PBAT_18	23.43	Confirmed	0.26	[0.18, 0.35]	0.04
PBAT_12	16.38	Confirmed	0.18	[0.09, 0.28]	0.02	PBAT_3	15.11	Confirmed	−0.12	[−0.20, −0.04]	0.01
PBAT_17	14.02	Confirmed	0.06	[−0.03, 0.14]	0.00	PBAT_7	13.17	Confirmed	0.13	[0.05, 0.21]	0.01
PBAT_7	11.94	Confirmed	0.11	[0.02, 0.20]	0.01	PBAT_17	12.75	Confirmed	0.12	[0.04, 0.20]	0.01
PBAT_6	11.32	Confirmed	−0.16	[−0.24, −0.07]	0.02	PBAT_6	11.17	Confirmed	−0.11	[−0.19, −0.03]	0.01
PBAT_3	10.11	Confirmed	−0.1	[−0.18, −0.02]	0.01	PBAT_12	10.58	Confirmed	0.10	[0.01, 0.19]	0.00
PBAT_9	10.09	Confirmed	0.06	[−0.02, 0.14]	0.00	PBAT_8	10.24	Confirmed	−0.06	[−0.14, 0.03]	0.00
PBAT_13	9.17	Confirmed	−0.11	[−0.20, −0.02]	0.01	PBAT_13	7.4	Confirmed	−0.12	[−0.21, −0.03]	0.01
PBAT_10	7.7	Confirmed	0.01	[−0.08, 0.10]	0.00	PBAT_10	6.01	Confirmed	0.02	[−0.06, 0.11]	0.00
PBAT_2	6.43	Confirmed	0.05	[−0.04, 0.13]	0.00	PBAT_9	5.78	Confirmed	−0.02	[−0.10, 0.05]	0.00
PBAT_14	6.37	Confirmed	0.11	[0.02, 0.19]	0.01	PBAT_14	5.16	Confirmed	0.12	[0.04, 0.21]	0.01
PBAT_8	5.67	Confirmed	0.04	[−0.06, 0.13]	0.00	PBAT_2	4.71	Confirmed	−0.01	[−0.09, 0.07]	0.00
PBAT_5	5.05	Confirmed	−0.13	[−0.20, −0.06]	0.02	PBAT_16	3.01	Confirmed	−0.03	[−0.11, 0.05]	0.00
PBAT_16	3.01	Confirmed	0.00	[−0.09, 0.08]	0.00	PBAT_5	2.31	Tentative	−0.11	[−0.18, −0.05]	0.01
PBAT_1	2.81	Confirmed	−0.02	[−0.09, 0.06]	0.00	PBAT_15	2.21	Rejected	−0.02	[−0.10, 0.07]	0.00
PBAT_15	2.66	Tentative	0.01	[−0.08, 0.10]	0.00	PBAT_1	2.18	Rejected	0.01	[−0.07, 0.08]	0.00
PBAT_11	1.94	Rejected	0.02	[−0.06, 0.10]	0.00	PBAT_11	0.35	Rejected	0.04	[−0.04, 0.12]	0.00
PBAT_4	0.58	Rejected	0.00	[−0.08, 0.07]	0.00	PBAT_4	−0.47	Rejected	−0.01	[−0.08, 0.06]	0.00

Note: GAD-7—Generalized Anxiety Disorder-7, PHQ-9—Patient Health Questionnaire-9, PBAT—Process-Based Assessment Tool, meanImp—mean importance, 95% CI = −95% coefficient interval, LL—lower limit of confidence interval, UL—upper limit of confidence interval, *sr*^2^—squared semi-partial correlation, *R*^2^—coefficient of determination. ** *p* < 0.01.

## Data Availability

The data presented in this study are available on request from the corresponding author due to participant privacy protection, given the sensitive nature of provided information.

## Supplementary Materials

The following supporting information can be downloaded at: https://www.mdpi.com/article/10.3390/bs15121697/s1.

## Abbreviations

The following abbreviations are used in this manuscript:

EEMM	Extended Evolutionary Meta-Model
GAD-7	Generalized Anxiety Disorder-7
PBAT	Process-Based Assessment Tool
PBT	Process-Based Therapy
PHQ-9	Patient Health Questionnaire-9

## Appendix A

### Appendix A.1. The English Version of the PBAT (Original)

Please mark on the line how much you agree with each statement. Base these responses on how you have been acting in the last week. Remember, there are no right or wrong answers.

0	10	20	30	40	50	60	70	80	90	100
Strongly disagree									Strongly agree	

1. I was able to change my behavior, when changing helped my life.										
0	10	20	30	40	50	60	70	80	90	100
2. I did things that hurt my connection with people who are important to me.										
0	10	20	30	40	50	60	70	80	90	100
3. I was able to experience a range of emotions appropriate to the moment.										
0	10	20	30	40	50	60	70	80	90	100
4. I struggled to keep doing something good for me.										
0	10	20	30	40	50	60	70	80	90	100
5. I did not find a meaningful way to challenge myself.										
0	10	20	30	40	50	60	70	80	90	100
6. I acted in ways that helped my physical health.										
0	10	20	30	40	50	60	70	80	90	100
7. My thinking got in the way of things that were important to me.										
0	10	20	30	40	50	60	70	80	90	100
8. I paid attention to important things in my daily life.										
0	10	20	30	40	50	60	70	80	90	100
9. I did things only because I was complying with what others wanted me to do.										
0	10	20	30	40	50	60	70	80	90	100
10. I stuck to strategies that seemed to have worked.										
0	10	20	30	40	50	60	70	80	90	100
11. I found personally important ways to challenge myself.										
0	10	20	30	40	50	60	70	80	90	100
12. I felt stuck and unable to change my ineffective behavior.										
0	10	20	30	40	50	60	70	80	90	100
13. I used my thinking in ways that helped me live better.										
0	10	20	30	40	50	60	70	80	90	100
14. I struggled to connect with the moments in my day-to-day life.										
0	10	20	30	40	50	60	70	80	90	100
15. I did things to connect with people who are important to me.										
0	10	20	30	40	50	60	70	80	90	100
16. I chose to do things that were personally important to me.										
0	10	20	30	40	50	60	70	80	90	100
17. I acted in ways that hurt my physical health.										
0	10	20	30	40	50	60	70	80	90	100
18. I did not find an appropriate outlet for my emotions.										
0	10	20	30	40	50	60	70	80	90	100

### Appendix A.2. The Romanian Version of the PBAT

Vă rugăm să indicați măsura în care sunteți de acord cu fiecare afirmație. Pentru a răspunde, gândiți-vă la ultima săptămână. Vă rog să luați în considerare că nu există răspunsuri corecte sau greșite. Utilizați scala de mai jos pentru a răspunde și încercuiți/ marcați locul unde vă încadrați cel mai bine:

0	10	20	30	40	50	60	70	80	90	100
Dezacord puternic									Acord puternic	

1. Am reușit să-mi schimb comportamentul, atunci când schimbarea a adus unele beneficii în viața mea.										
0	10	20	30	40	50	60	70	80	90	100
2. Am făcut lucruri care au influențat negativ legătura mea cu oamenii importanți pentru mine.										
0	10	20	30	40	50	60	70	80	90	100
3. Am avut capacitatea de a experimenta emoții adecvate în raport cu momentul trăit.										
0	10	20	30	40	50	60	70	80	90	100
4. M-am zbătut pentru a continua să fac lucrurile ce mi-au adus beneficii personale.										
0	10	20	30	40	50	60	70	80	90	100
5. Nu am avut în viața mea situații importante pe care să le consider o adevarată provocare.										
0	10	20	30	40	50	60	70	80	90	100
6. Comportamentul meu a fost adecvat în privința menținerii sănătății mele fizice.										
0	10	20	30	40	50	60	70	80	90	100
7. Felul în care am gândit în diferite situații m-a împiedicat să realizez lucruri importante pentru mine.										
0	10	20	30	40	50	60	70	80	90	100
8. Am fost atent (ă) la lucrurile pe care le-am considerat importante în viața mea de zi cu zi.										
0	10	20	30	40	50	60	70	80	90	100
9. Am făcut unele lucruri doar pentru că alții așteptau asta de la mine.										
0	10	20	30	40	50	60	70	80	90	100
10. Am rămas blocat (ă) la strategii care păreau utile în trecut.										
0	10	20	30	40	50	60	70	80	90	100
11. M-am ambiționat folosind diferite modalități personale.										
0	10	20	30	40	50	60	70	80	90	100
12. M-am simțit blocat (ă) și incapabil (ă) să-mi schimb un comportament pe care l-am considerat ineficient.										
0	10	20	30	40	50	60	70	80	90	100
13. Mi-am folosit gândirea pentru a reuși să îmi îmbunătățesc unele aspecte ale vieții.										
0	10	20	30	40	50	60	70	80	90	100
14. Am făcut eforturi pentru a mă conecta la momentele zilnice ce apar în viața mea.										
0	10	20	30	40	50	60	70	80	90	100
15. M-am străduit pentru a mă conecta cu oamenii pe care îi consider importanți pentru mine.										
0	10	20	30	40	50	60	70	80	90	100
16. Am ales să fac lucrurile ce au avut o anumită semnificație personală pentru mine.										
0	10	20	30	40	50	60	70	80	90	100
17. Am adoptat comportamente ce au dăunat sănătății mele fizice.										
0	10	20	30	40	50	60	70	80	90	100
18. Nu am găsit modalitatea potrivită prin care mi-aș putea exprima emoțiile.										
0	10	20	30	40	50	60	70	80	90	100

## Appendix B. Correspondence Between the PBAT Items Labels and Original Descriptions

**Item Number**	**Item Label**	**Original Item**
PBAT_1	AbleToChangeBehavior	I was able to change my behavior, when changing helped my life.
PBAT_2	HurtConnect	I did things that hurt my connection with people who are important to me.
PBAT_3	ExperienceRangeEmotions	I was able to experience a range of emotions appropriate to the moment.
PBAT_4	StruggledToKeepDoing	I struggled to keep doing something good for me.
PBAT_5	NoMeaningfulChallenge	I did not find a meaningful way to challenge myself.
PBAT_6	HelpHealth	I acted in ways that helped my physical health.
PBAT_7	ThinkingGotInWay	My thinking got in the way of things that were important to me.
PBAT_8	PaidAtToImportant	I paid attention to important things in my daily life.
PBAT_9	Complying	I did things only because I was complying with what others wanted me to do.
PBAT_10	StuckToStrategies	I stuck to strategies that seemed to have worked.
PBAT_11	ImportantChallenge	I found personally important ways to challenge myself.
PBAT_12	StuckUnableChange	I felt stuck and unable to change my ineffective behavior.
PBAT_13	ThinkingHelpedLife	I used my thinking in ways that helped me live better.
PBAT_14	StruggleConnectMoments	I struggled to connect with the moments in my day-to-day life.
PBAT_15	ConnectToPeople	I did things to connect with people who are important to me.
PBAT_16	PersonalImport	I chose to do things that were personally important to me.
PBAT_17	HurtHealth	I acted in ways that hurt my physical health.
PBAT_18	NoOutletForFeelings	I did not find an appropriate outlet for my emotions.
Note: Adapted after (Ciarrochi et al., 2022b).		
